# Low-cost prototype for bearing failure detection using Tiny ML through vibration analysis

**DOI:** 10.1016/j.ohx.2025.e00658

**Published:** 2025-05-22

**Authors:** Andres Felipe Cotrino Herrera, Jesús Alfonso López Sotelo, Juan Carlos Blandón Andrade, Alonso Toro Lazo

**Affiliations:** a School of Engineering and Basic Sciences, Universidad Autónoma de Occidente, Cali, Colombia; b Systems and Telecommunications Engineering Program, Universidad Católica de Pereira, Pereira, Colombia

**Keywords:** Artificial intelligence, Machine learning, Teaching strategy, Vibration analysis

## Abstract

The document presents a low-cost, open-source device designed to facilitate the learning of technologies like artificial intelligence in embedded systems through vibration analysis. It also aims to enhance students’ skills by introducing industrial challenges into the classroom via a scaled-down prototype. This study analyzes the vibrations generated by bearings to classify, using Artificial Intelligence (AI), whether they are defective. The device integrates electronic, mechanical, and software components, leveraging online technologies and platforms like Arduino to support hands-on learning. The document provides detailed instructions on the components used, circuit connections, step-by-step construction, and implementation, allowing replication of the prototype. This device fosters the development of STEM skills, promotes the application of AI and TinyML in real-world contexts, and enriches educational programs by encouraging interdisciplinary learning.

## Graphical abstract

**Figure:**
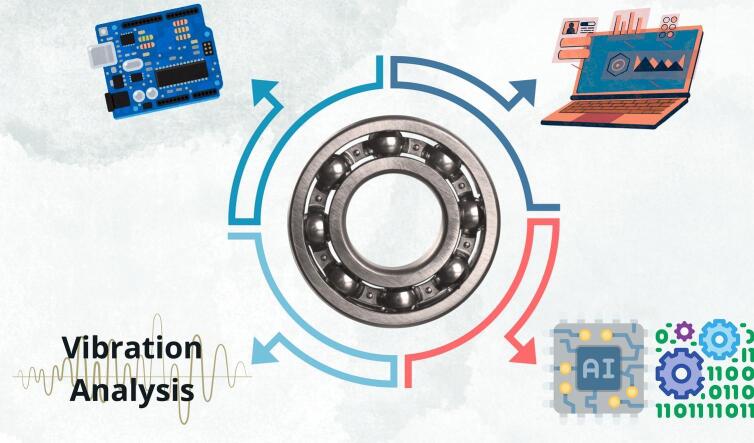

Specifications table.

**Table:** 

Hardware name	Prototype for analyzing vibrations produced by a bearing for a DC motor using Machine Learning
Subject area	• Engineering and materials science • Educational tools and open-source alternatives to existing infrastructure
Hardware type	• Electrical engineering and computer science • Mechanical engineering and materials science • Artificial Intelligence
Closest commercial analog	CMCP601 Demonstration Rotor Kit
Open-source license	GNU General Public License (GPL) 3.0
Cost of hardware	US $270.7
Source file repository	https://doi.org/10.17632/685hm7n8nb.1

## 1 Hardware in context

Vibration analysis in engines has become very important nowadays due to the constant advances in predictive maintenance. It is more feasible to analyze plant elements' behavior than to wait for a failure to occur. These techniques allow for noninvasive or nondestructive analysis of machines to predict when they require part replacements or repairs [1].

For the development of this prototype, we focused on bearings, as most electrical machines use this type of rotating element. Even under normal scenarios, vibrations can vary depending on the type of failure the bearing has, such as loss of lubrication, wear, missing balls inside, and notches on the raceway (brinelling), among others [2]. Although many studies today have focused on vibration analysis in motors, most do so for AC motors, simulating bearing failures and saving the data to train models that allow future predictions [3]. On the other hand, research on DC motors employs deep learning algorithms, which have a high consumption of computational resources to achieve correct detection (whether or not a failure is present) [4]. They also implement derivatives of the Kalman filter, to which they input the vibrations produced by the rotating element and other plant variables (phase current) to process the data and obtain an accurate result [5].

In the field of artificial intelligence AI, studies have also been conducted to analyze the vibration obtained from a bearing and classify whether it has any failures using artificial neural network (ANN) and support vector machine (SVM) models, resulting in both being quite effective [6]. Alternatively, research has been conducted to analyze the vibrations of bearings using the empirical mode decomposition method (EMD), extracting the most important features to subsequently use a classifier to identify whether there is a failure or not [7]. Notably, in industrial environments, vibration measurements are inevitably mixed with noise, which has led to research using (AI) methods based on denoising autoencoders (DAE) to remove the noise and retain important data for signal processing, feature extraction and obtaining classification results [8].

The role of vibration monitoring in predictive maintenance cannot be overstated. With higher demands for plant efficiency and the complexity of modern machinery, a well-structured maintenance strategy that includes vibration analysis helps detect early signs of equipment deterioration, allowing for planned maintenance and reducing unexpected failures. Monitoring critical components like bearings can predict potential issues, minimizing downtime and repair costs, ultimately enhancing plant productivity and operational efficiency [9].

Other prototypes or products on the market allow for some tasks, such as vibration analysis. For example, the CMCP601 Demonstration Rotor Kit is a test bench for a motor designed to realistically simulate the vibrations produced by a plant [10].

This prototype provides a tool for simulating industrial elements and failures at the academic level. Due to the constant development of TinyML (Tiny Machine Learning), we aim to promote the use of AI models to solve problems such as vibration analysis [11].

Additionally, all types of AI, such as machine learning, are gaining increasing importance in all areas of higher education today. AI is used to improve teaching and learning, create enhanced educational experiences, streamline processes, and accelerate academic research [12].

Currently, it is very common for academic programs that encourage this type of technology in embedded systems to conduct practices with common sensors (microphone, camera, accelerometers, and gyroscopes, among others) that do not go beyond creating a simple dataset and an AI model to give a glimpse of the great potential of this field. Based on this, we decided to offer the opportunity to bring the industry into the academic environment and simulate these analyses as if it were a real plant.

Additionally, low-cost construction materials, such as PLA (polylactic acid) and acrylic, were chosen to make the prototype replicable and implementable in various courses that encourage these technologies. Related to the other components like inertial sensors, Arduino BLE, and mechanical elements (shafts, bearings, flexible couplings), these allow students to develop their skills, enhance their knowledge, and familiarize themselves with what they might encounter in an industrial environment.

Ultimately, the above will foster the use and applications of AI and embedded devices (TinyML) and develop STEM skills (Science, Technology, Engineering, Mathematics).

## 2 Hardware description

**Fig. 1:**
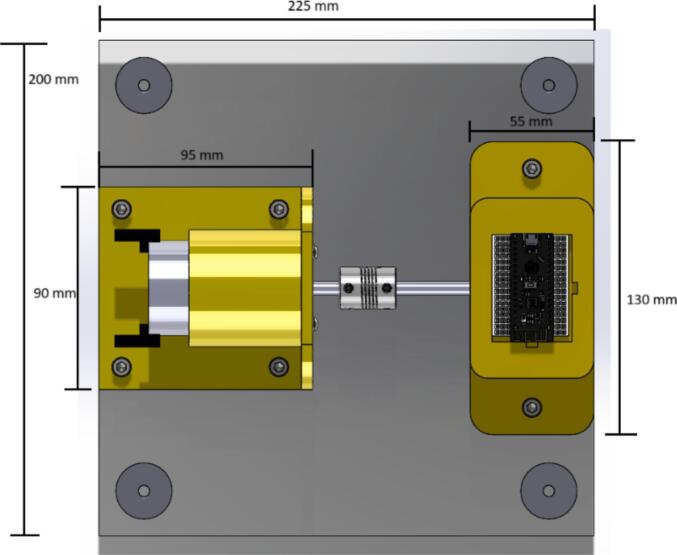
Dimensions.

This prototype operates due to a DC motor connected to a variable 24-volt power supply. The motor's rotation is transmitted through a shaft, utilizing a flexible coupling that connects the motor shaft to the bearing shaft. Once the power supply is turned on, the bearing starts to rotate, producing vibrations in the setup, where an inertial sensor can be attached (our prototype allows the connection of different types of sensors) for the respective analysis. By collecting data from bearings in good condition or with defects, a Machine Learning model can be created to classify the vibrations between a bearing with or without defects. Fig. 1 shows the general concept of the prototype.

The classification model was developed using the Edge Impulse platform, which is specifically designed to support embedded devices such as Arduino, Raspberry Pi, and Jetson, among others. This platform is freely accessible and enables the complete workflow—from data acquisition and model creation to training and deployment—on the target device for effective vibration classification. A major advantage of this approach is that, by allowing the deployment of the trained model directly onto the embedded system, users can display the classification results (i.e., bearing in good condition or with defects) through actuators, screens, or visual indicators.

The developed prototype has two important subsystems or parts: mechanical and electronic.

### 2.1 Mechanical part

The mechanical design was created to obtain a portable and low-cost device; therefore, it is 3D printed, and its dimensions are suitable for easy transport. Fig. 1 shows an acrylic base of 225 mm × 200 mm × 3 mm, which supports each of the elements of the setup. The base for the motor and the bearings are designed to be manufactured using 3D printing with PLA material at a thickness of 1.75 mm, so these pieces do not take up much space nor require much material to be printed.

### 2.2 Electronic part

**Fig. 2:**
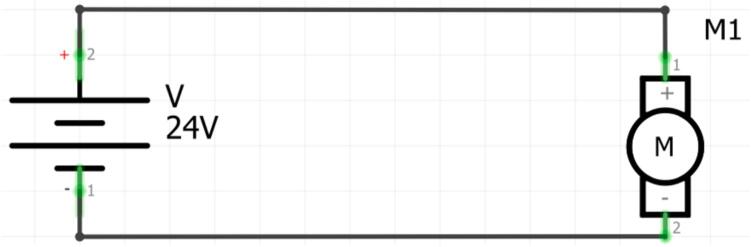
Connection diagram for DC Motor.

**Fig. 3:**
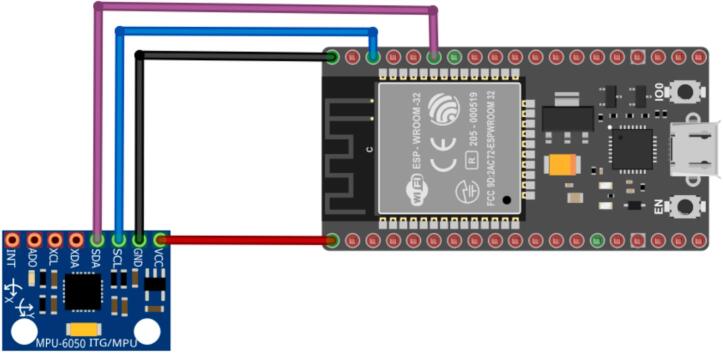
ESP32 and MPU6050 connection.

The entire system is powered by a 24 Vdc power supply, which energizes the motor. However, as mentioned earlier, there is the possibility of connecting different types of inertial sensors; for example, tests were conducted with the MPU6050, the integrated IMU of the Arduino BLE Sense Rev2 (BMI270), and the Arduino BLE Sense (LSM9DS1) [13]. Depending on the connected sensor, the implementation of the connection diagram will vary. For Arduino, only a USB cable is needed to transfer data to Edge Impulse, while for the MPU6050, a board like the ESP32 is necessary to send the data collected by the sensor via the client's program. Fig. 2, Fig. 3 below show the motor and the MPU6050 connection diagrams.

Through this development, interested parties such as students and professors related to artificial intelligence will be able to delve deeper into various concepts, put learned theory into practice, and, most importantly, understand on a small scale the problems that can arise in industries. Additionally, the fact that the prototype includes mechanical and electronic design concepts provides the opportunity to integrate different engineering disciplines or promote others, such as mechatronics. Furthermore, it promotes learning in cutting-edge topics such as artificial intelligence and machine learning, thereby increasing interest in TinyML and showcasing the significant capabilities of embedded devices in developing industrial applications or AI models.

For all interested parties, this device can help with:

• Simulating industrial processes on a small scale.
• Recreating scenarios of vibration analysis and bearing failures.
• Learning concepts of artificial intelligence and machine learning.
• Promoting the use of embedded devices to implement AI models.

## 3 Design files summary

The designed files for the device are presented below in Table 1, and a brief description of each will be provided.

**Table 1:** Design files summary.

**Design file name**	**File type**	**Open-source license**	**Location of the file**
AcrylicBase.SLDPRT	CAD	GNU GPL v3	Available with the article
BearingBase1.SLDPRT	CAD	GNU GPL v3	Available with the article
BearingBase2.SLDPRT	CAD	GNU GPL v3	Available with the article
MotorBase.SLDPRT	CAD	GNU GPL v3	Available with the article
AcrylicBase.DXF	DXF	GNU GPL v3	Available with the article
BearingBase1.STL	STL	GNU GPL v3	Available with the article
BearingBase2.STL	STL	GNU GPL v3	Available with the article
MotorBase.STL	STL	GNU GPL v3	Available with the article
esp32IMU.txt	txt	GNU GPL v3	Available with the article

**AcrylicBase.SLDPRT:** The file was designed to cut the acrylic sheet to the necessary size.

**BearingBase1.SLDPRT:** The file is designed to support the bottom part of the bearing and serve as the main base to be attached to the acrylic base.

**BearingBase2.SLDPRT:** The file is designed to support the top part of the bearing and holds the protoboard for connecting the inertial sensors.

**MotorBase.SLDPRT:** The file was designed to support the DC motor in a fixed position on the acrylic base.

**BearingBase1.STL:** File in format for 3D printing.

**BearingBase2.STL:** File in format for 3D printing.

**MotorBase.STL:** File in format for 3D printing.

**esp32IMU.txt:** Text file with code for reading IMU data for Arduino IDE.

## 4 Bill of materials summary

All prices presented below in Table 2, Table 3 are in US dollars.

**Table 2:** Bill of materials summary.

**Component**	**Number**	**Cost per number**	**Total cost**	**Material type**
DC Motor Crouzet 24v (42 mm diameter – 6 mm diameter shaft)	1	133.37	133.37	Other
Acrylic sheet (300 mm × 300 mm × 3 mm)	1	5.92	5.92	Polymer
Steel Axle (6 mm diameter × 1 m)	1	5.51	5.51	Metal
3D printing material PLA (1 kg)	1	20.05	20.05	Polymer
Flexible Coupling 6 mm × 6 mm	1	2.51	2.51	Metal
Bearing 626ZZ (6 × 19 × 6 mm)	2	1.50	3.00	Metal
M3 screw × 10 mm	2	0.05	0.10	Metal
M3 flat washer	2	0.025	0.05	Metal
M5 screw * 15 mm	10	0.14	1.40	Metal
M5 nut	10	0.088	0.88	Metal
The base supports (20 mm diameter)	4	0.25	1.00	Polymer
USB Cable for Arduino	1	3.00	3.00	Other
Mini protoboard (35 × 47 × 8.5 mm)	1	1.63	1.63	Polymer
**Total cost of materials (US$)**			**178.42**

**Table 3:** Additional materials.

**Component**	**Description**	**Quantity**	**Cost per unit**	**Total Cost**
ESP32	ESP32 development board	1	8.77	8.77
Arduino BLE Sense	Arduino Development Board with IMU sensor integrated (LSM9DS1)	1	40.50	40.50
Arduino BLE Sense Rev2	Arduino Development Board with IMU sensor integrated (BMI270)	1	40.50	40.50
Wires	200 mm / M to F / 10 pz	1	2.51	2.51
**Total cost of additional materials (US$)**			**92.28**	

Additionally, Table 3 presents the additional materials (sensors and related materials used to validate the bank's operation).

## 5 Build instructions

The following are the steps to construct the device:

1. Perform the 3D printing of the files with the “.STL” extension (located in section 3). After printing, verify that the pieces fit correctly together; otherwise, post-processing is necessary, depending on the quality of the finished piece (sanding some walls and removing supports).
2. Upload the file “AcrylicBase.DXF” to a laser cutter and place the acrylic piece to make the respective holes to secure the test bench later.
3. Cut the steel shaft using a saw to obtain two smaller, 100 mm long shafts.
4. Prepare all components, such as screws, nuts, and washers, for assembling the parts.
5. Insert the DC motor into the 3D printed piece called “MotorBase.STL,” aligning the holes on the front of the motor with those on its base. Once correctly positioned, place the M3 × 10 mm screws along with the washers. Fig. 4 provides a detailed view of the above-mentioned step.

**Fig. 4:**
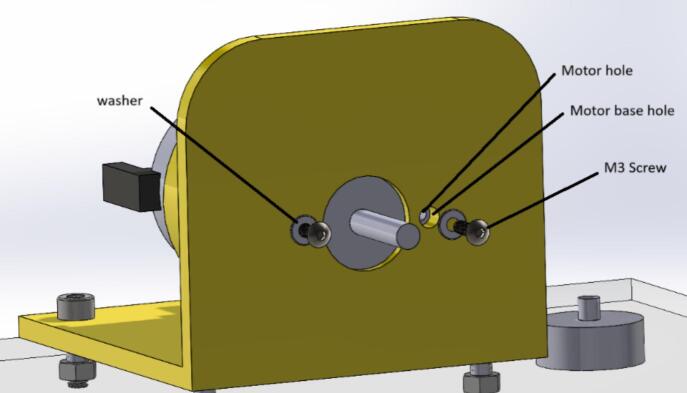
Build instructions for step 5.
6. Once the motor is secured to its base, it should be screwed to the acrylic using the four holes as a guide (see Fig. 5 for more details on positioning the piece). Use 4 M5 × 15 mm screws and their respective M5 nuts to secure them to the bottom of the acrylic (see Fig. 6).

**Fig. 5:**
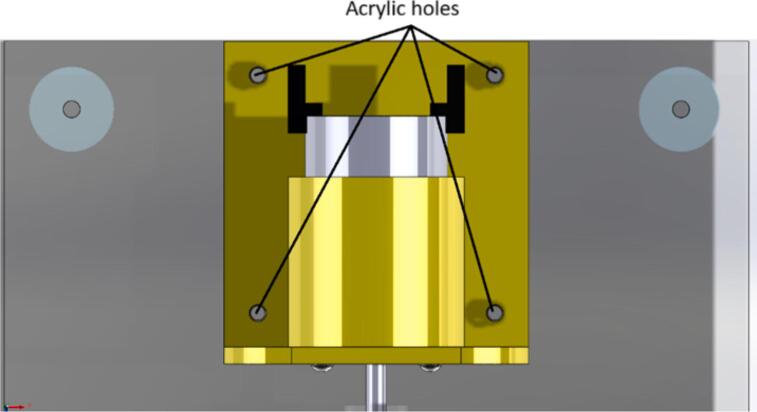
Build instructions for step 6.

**Fig. 6:**
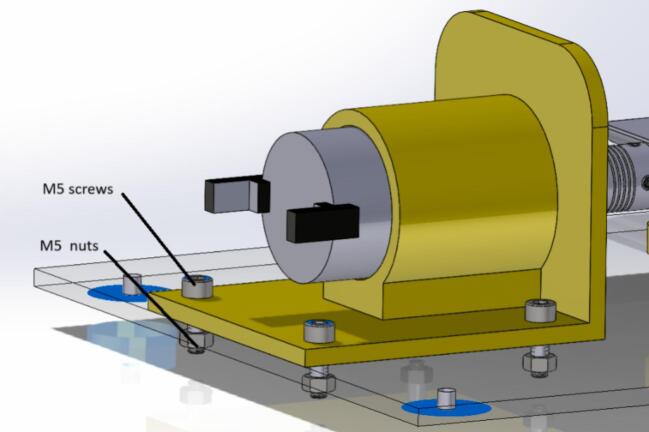
Build instructions for step 6.
7. Position the 3D printed piece called “BearingBase1.STL” on the acrylic to screw it in with two M5 × 15 mm screws and their respective nuts. See Fig. 7 for more information on positioning the piece for assembly.

**Fig. 7:**
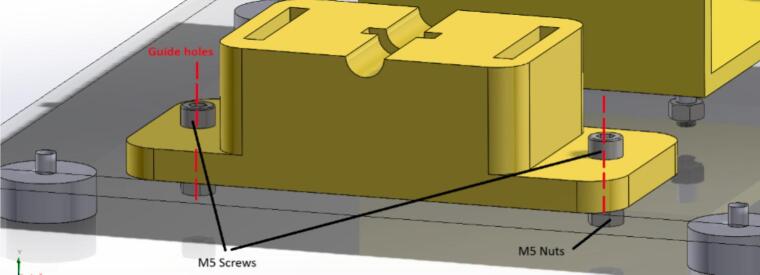
Build instructions for step 7.
8. Insert the flexible coupling into the motor shaft (see Fig. 8 for more detail on proper positioning). It leaves space for later securing it to the bearing shaft.

**Fig. 8:**
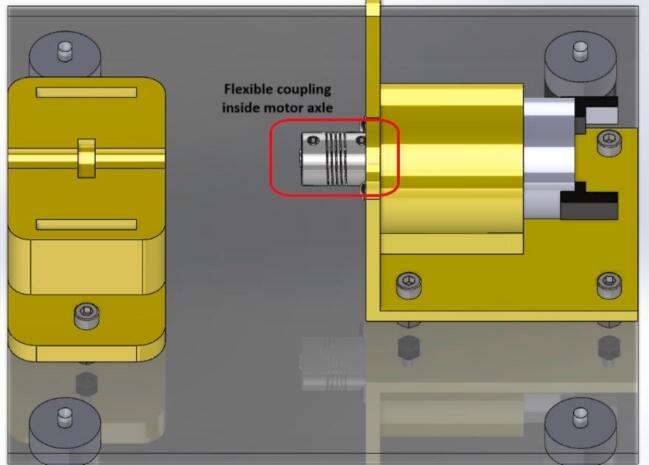
Build instructions for step 8.
9. Insert the 100 mm long steel shafts into the two bearings and place the latter in the designated space in the “BearingBase1.STL” piece. See Fig. 9 for more details on how to position the bearing.

**Fig. 9:**
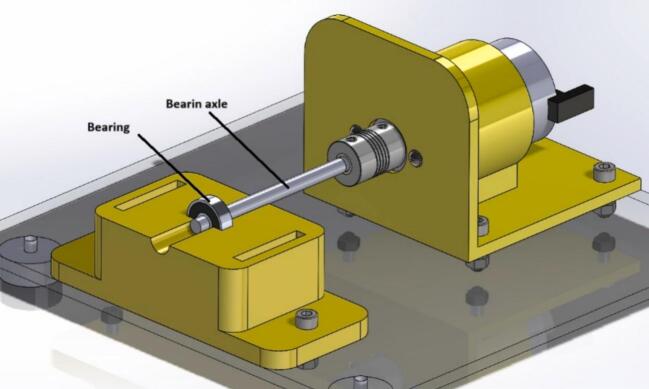
Build instructions for step 9.
10. Move the flexible coupling to position it between the motor shaft and the bearing shaft. Then, secure the coupling to the shafts with a 5/64″ Allen wrench using the four screws located on the coupling (see Fig. 10 for more detail).

**Fig. 10:**
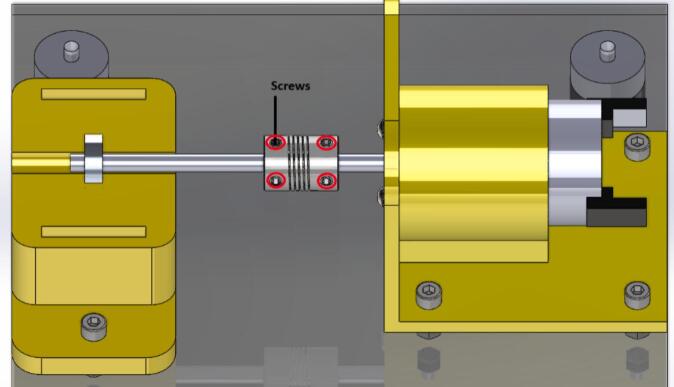
Build instructions for step 10.
11. Insert the mini protoboard into the top part of the 3D printed piece called “BearingBase2.STL,” using the shape of the component as a guide. Once done, position “BearingBase2” on top of “BearingBase1,” using the holes in the latter as a guide.
12. Place the four base supports using the four holes located at the corners of the test bench as a guide and secure them with the remaining four M5 * 15 mm screws and nuts.
13. The user can connect their preferred sensor to the mini protoboard.

**Fig. 11:**
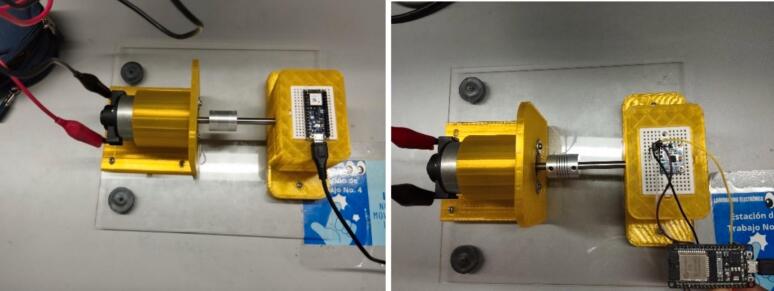
Finished physical device.

The finished physical device should look like the one shown in Fig. 11.

### 5.1 Bearing replacement

1. Using the 5/64″ Allen wrench, loosen the coupling screws to release both shafts. Push the flexible coupling back completely onto the motor shaft, freeing the bearing shaft (see Fig. 8).
2. Carefully loosen the top cover of the bearing support (BearingBase2). This will allow you to release the entire bearing with its shaft.
3. Once the space is free, place the other bearing (defective or non-defective, as the case may be) to continue data collection.
4. Reposition the flexible coupling between both shafts (bearing and motor) to secure them (follow step 10, shown earlier in this section).

## 6 Operation instructions

**Fig. 12:**
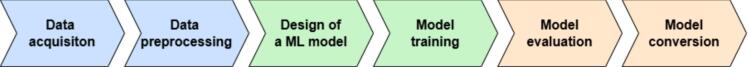
Workflow for operation.

A specific workflow must be followed to properly operate the device, encompassing everything from data collection to model conversion and validation testing. A schematic diagram outlines the step-by-step procedure for operating the prototype (see Fig. 12).

It is important to highlight that the Edge Impulse platform is employed as the primary tool to carry out each of these steps successfully. The following section outlines the operational instructions that must be followed to comply with the previously presented workflow. In this context, the subsequent points pertain to data acquisition and preprocessing.

1. Connect the DC motor to the 24-volt power supply using alligator clips to secure the device's connection tabs (see Fig. 13).

**Fig. 13:**
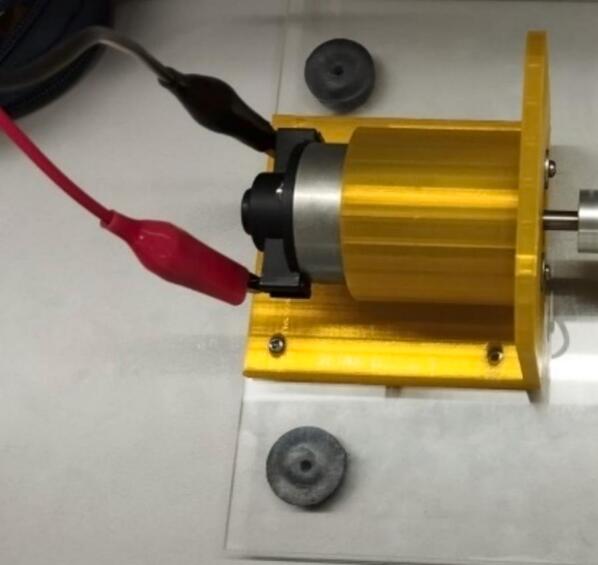
Operation instructions for step 1.
2. Connect the desired inertial sensor, either MPU6050 (see Fig. 3 for connection schematic) or one of the Arduinos (BLE Sense or BLE Sense Rev 2) via USB directly to the computer.
3. Once the connections for the power supply and the sensor are ready, turn on the power supply and start collecting data.
4. Access Edge Impulse online via the link: https://edgeimpulse.com/
5. Log in on the homepage using the “Log In” section. Create one if you do not have an account (see Fig. 14, Fig. 15).

**Fig. 14:**
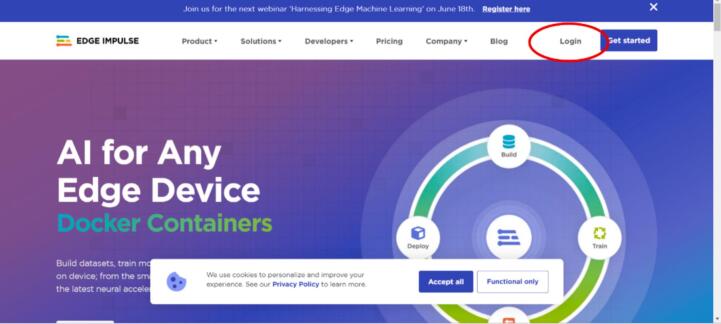
Operation instructions for step 5.

**Fig. 15:**
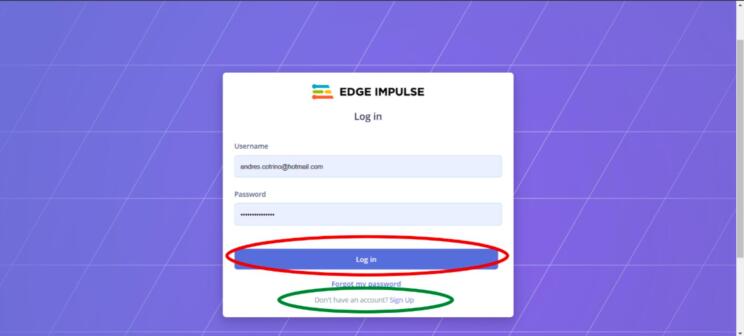
Operation instructions for step 5.
6. Once logged in, click the “Create new Project” option (see Fig. 16). Name the project and proceed to the next step.

**Fig. 16:**
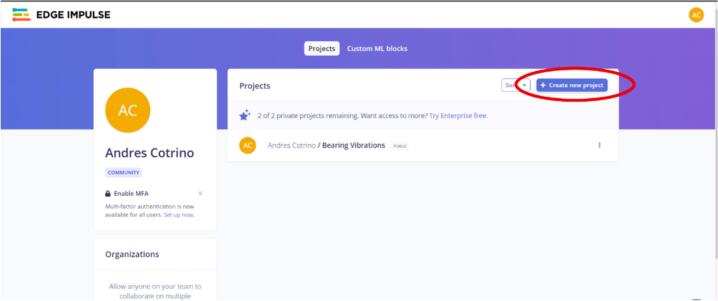
Operation instructions for step 6.
7. In the project's main menu, click “Data acquisition” on the left side of the screen (see Fig. 17). At this point, you must follow different steps to connect your chosen sensor to Edge Impulse. The steps for each sensor are detailed below.

**Fig. 17:**
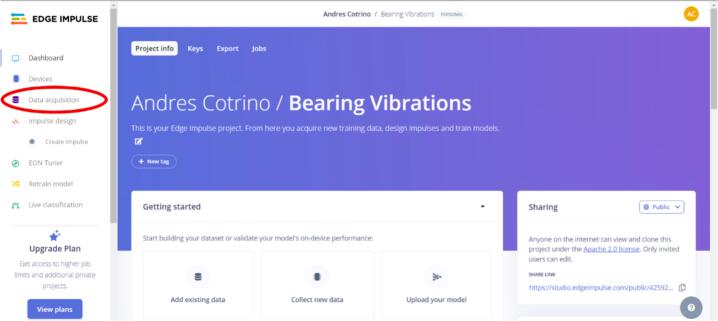
Operation instructions for step 7.

### 6.1 Arduino BLE sense or arduino BLE sense rev 2

**Fig. 18:**
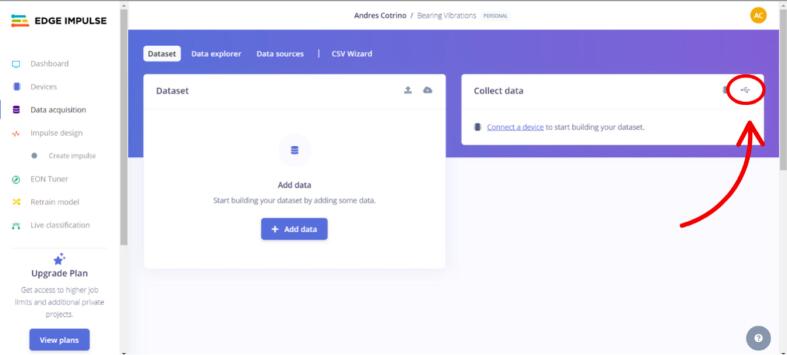
Operation instructions for Arduino connection.
c. In the pop-up tab that appears in the browser, click on the Arduino board (see Fig. 19).

a. Connect the Arduino of your choice via USB.
b. In the “Data acquisition” tab, specifically under “Collect Data,” click on the USB connection icon (see Fig. 18 for more detail).

**Fig. 19:**
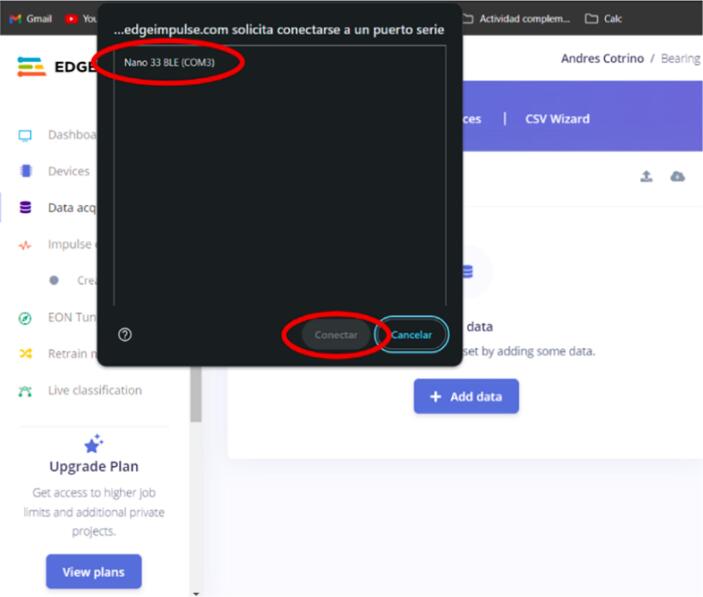
Operation instructions for Arduino connection.
d. Verify that the device connects correctly and that the different sensors available on these devices can be selected under “Collect Data” (see Fig. 20).

**Fig. 20:**
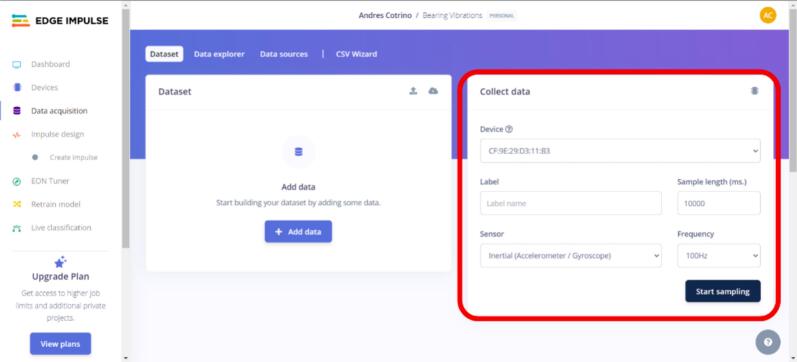
Operation instructions for Arduino connection.

### 6.2 MPU6050 + ESP32

**Fig. 21:**
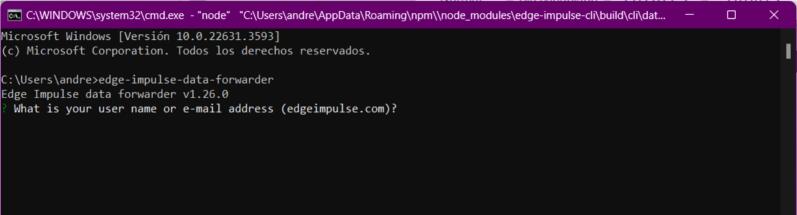
Operation instructions for MPU6050 connection.
f. Once logged in, the client will identify the device connected via the computer’s USB ports. If not, select it using the keyboard arrows. Once the device is connected, the client will prompt you to choose the project to send the data read from the sensor (see Fig. 22).

a. Using the Arduino IDE, upload the program to read data from the sensor and send it via serial. Paste the text from the “esp32IMU.txt” [14] (file located in section 3) into the Arduino IDE to upload it to the ESP32.
b. Once the code is uploaded to the board, check the wire connections and proceed to the next step.
c. Install the Edge Impulse client via the terminal using the following link: https://docs.edgeimpulse.com/docs/tools/edge-impulse-cli/cli-installation
d. Verify that the ESP32 is correctly connected via USB to the computer.
e. Once the Edge Impulse client is installed, run the following command in the computer terminal: “edge-impulse-data-forwarder.” Log in to your Edge Impulse account using your username/email and password (see Fig. 21).

**Fig. 22:**
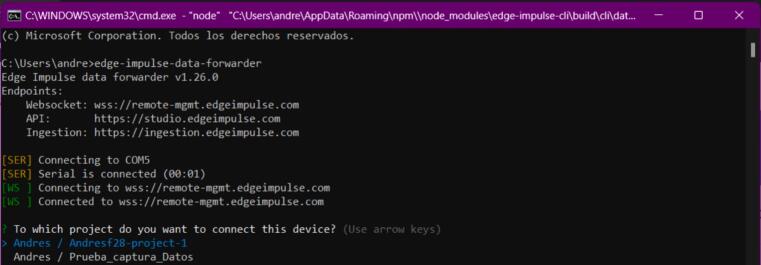
Operation instructions for MPU6050 connection.
g. When the client recognizes the data from the sensor, you will be asked to name each of the incoming variables. For this case, you should label the values as “ax,” “ay,” and “az” for the three acceleration axes, which are most relevant for this analysis, and name the device (see Fig. 23).

**Fig. 23:**
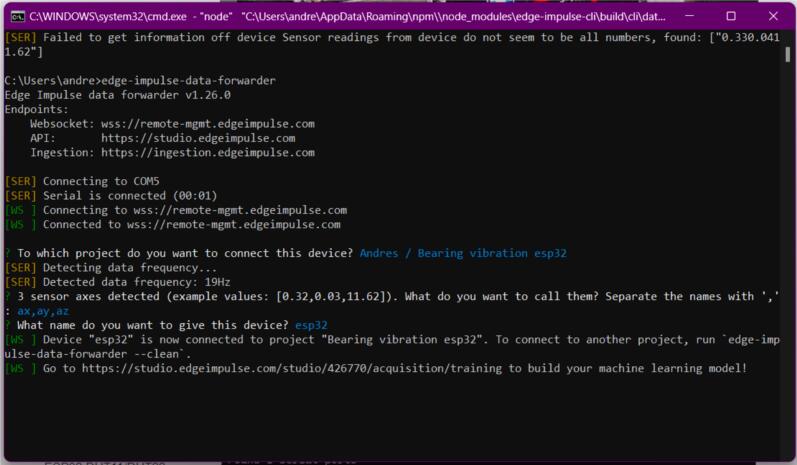
Operation instructions for MPU6050 connection.
h. After this step, go to the Edge Impulse website in the “Data Acquisition” section, where you can select the previously named device and choose the data to be sampled (see Fig. 24).
  8. Once the sensor is correctly connected, turn on the power supply and collect data by clicking the “start sampling” button. A sampling frequency of 20 Hz is recommended, and each analysis class should be labeled (in this case, bearings with and without defects). See Fig. 25 for more details.

**Fig. 25:**
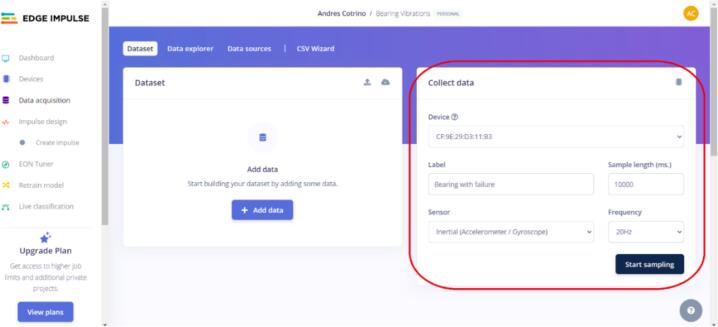
Operation instruction for step 8.
  9. Each class should take at least 15 to 20 samples. To change the bearing, follow the steps at the end of the previous section.

**Fig. 24:**
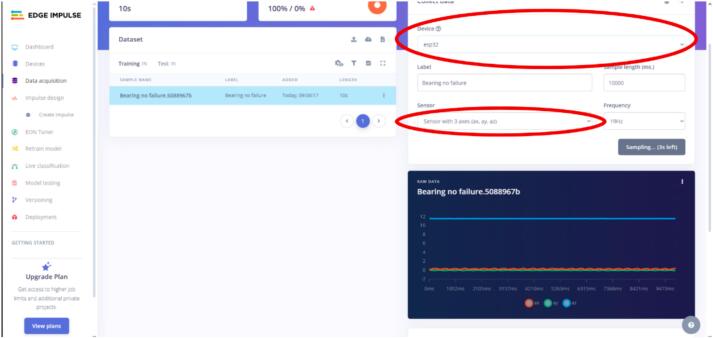
Operation instructions for MPU6050 connection.

Once the initial data collection stage has been completed, the process continues with creating and training a machine learning model, as illustrated in the diagram shown in Fig. 12.

A multilayer perceptron (MLP) neural network is utilized to classify the fault of bearings. This architecture is included by default within Edge Impulse for such applications. Before passing into the neural network, the input data undergoes a preprocessing step to extract spectral features. The Fast Fourier Transform (FFT) is the recommended method to enhance classification performance in vibration analysis and is therefore adopted in this project.

This process results in thirty-nine processed features, which corresponds to thirteen features per axis. Specifically, for each axis, three of these features belong to the time domain, comprising the statistical analyses of Root Mean Square (RMS), Skewness, and Kurtosis. The remaining features are associated with the frequency domain, where spectral analysis is performed to extract the maximum value from the Fast Fourier Transform (FFT) of each window generated from the collected data [15,16].

Once all the spectral features are extracted, they are fed into the MLP, which has an input layer of size thirty-nine (representing all extracted features), two dense layers (with 20 and 10 neurons, respectively), and an output layer with two neurons corresponding to the classification targets.

**Fig. 26:**
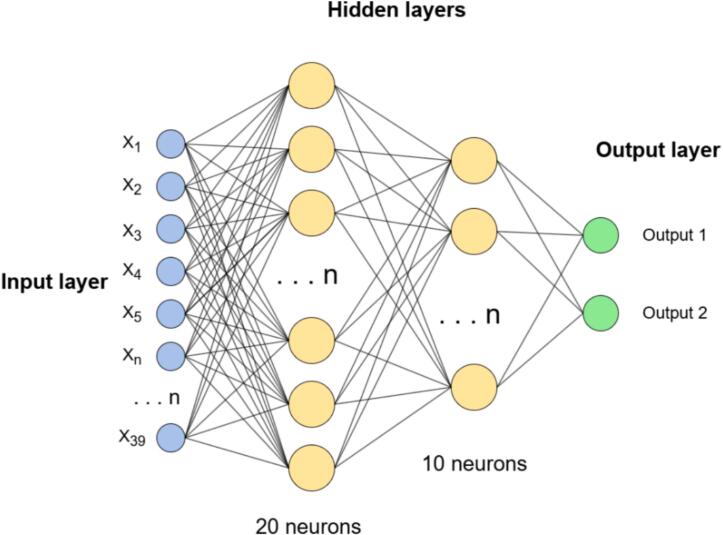
MLP neural network architecture.

Fig. 26 presents the diagram of the implemented neural network architecture.

The following section describes the steps required to design and train the model by the established workflow.

10. Switch to the “Impulse Design” tab once all the data is collected. This section selects a data processing block and a block to classify the classes. To process the data, click “Add a processing block” and select “Spectral Analysis.” In the resulting block, mark only the axes corresponding to acceleration, such as “accX, accY, and accZ.” For the “learning block,” click on it and select “Classification.” Click “Save impulse” (see Fig. 27 to verify the creation of the model).

**Fig. 27:**
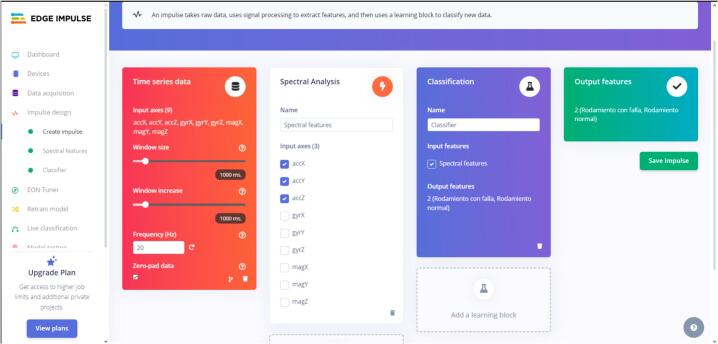
Operation instruction for step 10.
11. As shown in Fig. 27, two new tabs, “Spectral features” and “Classifier,” are created in the “Impulse design” section. Click on “Spectral features,” click “Save parameters,” and then “Generate Features” (see Fig. 28, Fig. 29).

**Fig. 28:**
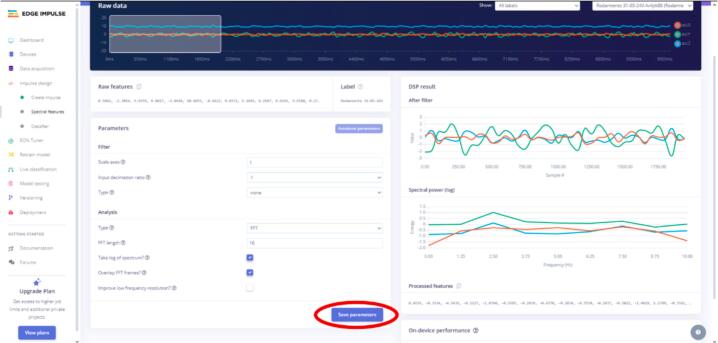
Operation instruction for step 11.

**Fig. 29:**
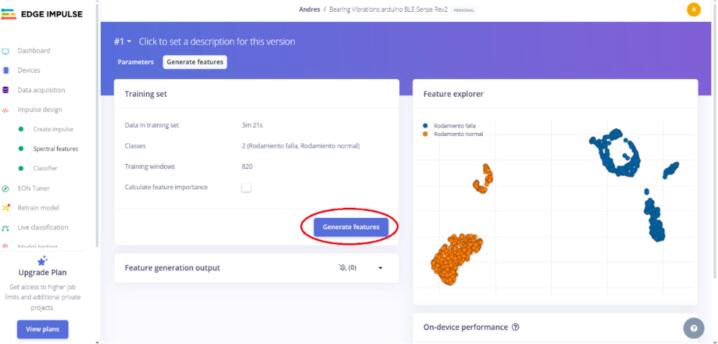
Operation instruction for step 11.
12. After completing the previous step, click the “Classifier” tab and “Start training” to train the model. See Fig. 30.

**Fig. 30:**
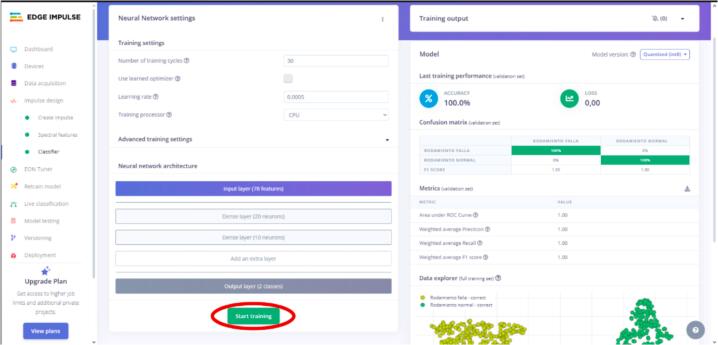
Operation instruction for step 12.

Once the model training is completed, the model conversion and evaluation stage concludes. The following steps are carried out to accomplish this.

13. Click on the “Deployment” section, and in “Search deployment options,” look for the device on which you want to run the classification model. The website offers many other options, including an Arduino library for ESP32. See Fig. 31 for more details.

**Fig. 31:**
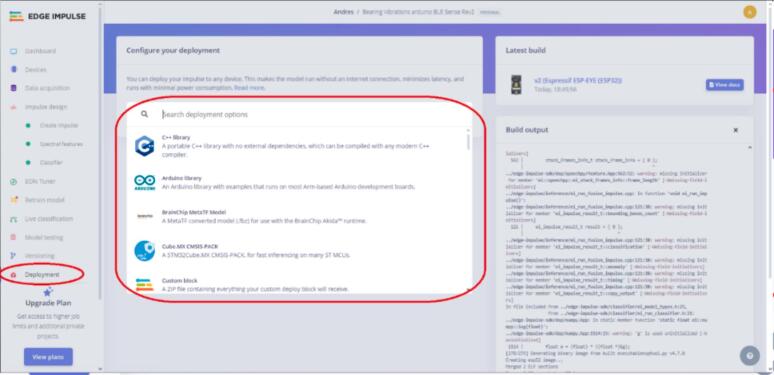
Operation instruction for step 13.
14. If you choose an Arduino library for deployment, go to the Arduino IDE and load the library. Once the library is correctly installed, go to the “File” tab, then “Examples,” and look for the file name generated by Edge Impulse when exporting the library. Then, select the board you are working with (in the example image, it will be the BLE Sense Rev 2) and the example file named “accelerometer_continuous.” See Fig. 32 for more details on the file to select. This way, the model will be uploaded to the chosen board, and the classification will run continuously if powered.

**Fig. 32:**
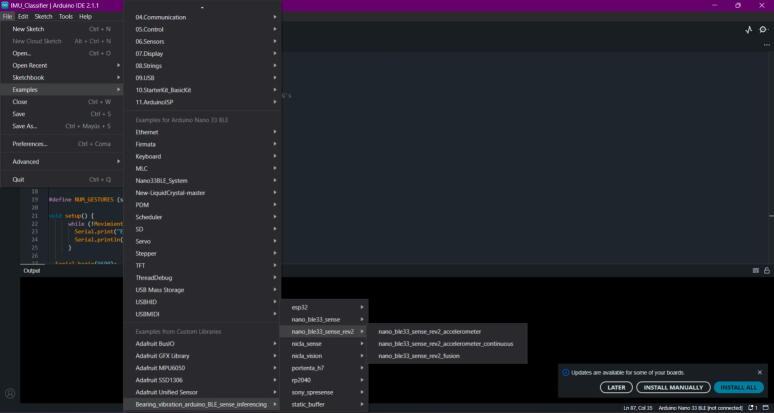
Operation instruction for step 14.

## 7 Validation and characterization

Technical specifications of the prototype are presented below in Table 4:

**Table 4:** Technical specifications.

**Technical aspect**	**Description**
Input voltage	24 V
Max. Power	22 W
Current	< 1 A
Prototype weight	1 kg
Material	Acrylic, PLA (Polylactic Acid) thickness 1.75 mm for 3D printing
Software	Edge Impulse

**Fig. 33:**
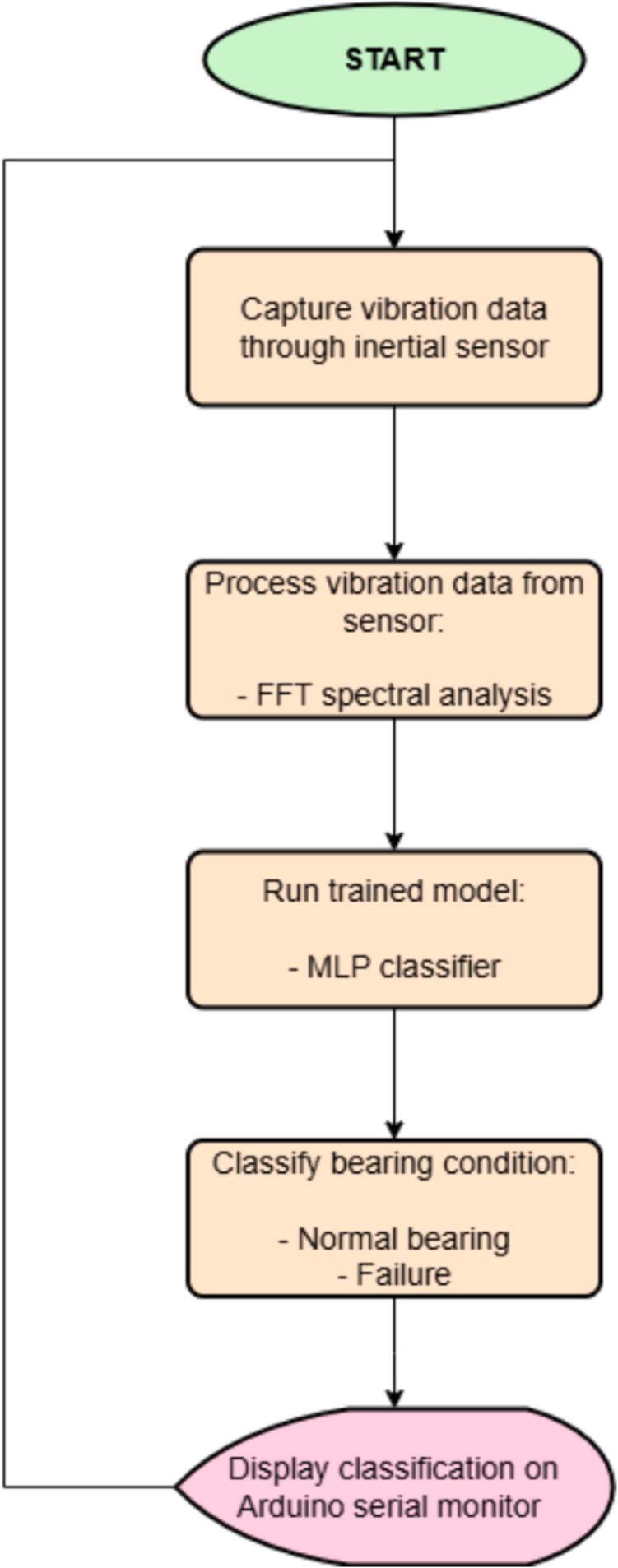
Software description: Model operation.

Based on Section 6, Fig. 33 presents a flow chart to illustrate the system’s operation regarding the implemented software. This diagram represents the sequence of operations executed after the model is deployed on the Arduino board.

According to the diagram, the software implemented on the Arduino platform is responsible for several tasks during this model testing stage. The first one is to acquire vibration data through inertial sensors. Three sensors were used: Arduino Nano 33 BLE Sense, Nano 33 BLE Sense Rev 2, and an MPU6050 module connected to an ESP32.

**Fig. 34:**
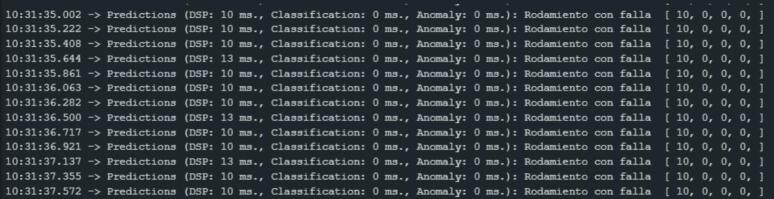
Deployment results.

Once the vibration data is acquired, the software processes the input through FFT spectral analysis and feeds the resulting values into the deployed model. The model then classifies each sample captured by the sensor. The classification result (good bearing or faulty one) is displayed in real time via the Arduino IDE serial monitor. Fig. 34 shows the output data as displayed on the serial monitor.

Tests were performed before deploying the model (as shown above) using the website's “Live Classification” feature. This feature allows real-time data collection and subsequent classification of the results based on the previously trained model, and it is highly useful for validating the model's correct functioning before deploying it on a device such as Arduino.

**Fig. 35:**
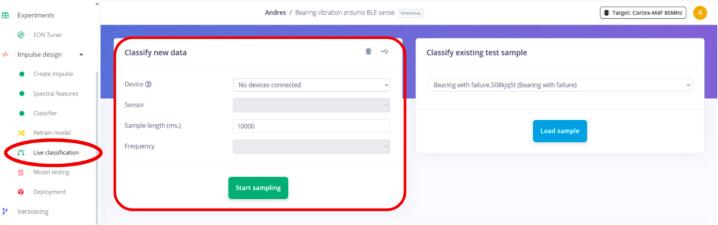
Live classification on Edge Impulse.

Once the model has been trained, this test can be performed by accessing the “Live Classification” tab in Edge Impulse (see Fig. 35). Upon connecting the sensor, the platform will be ready to capture data and perform the corresponding classification.

**Fig. 36:**
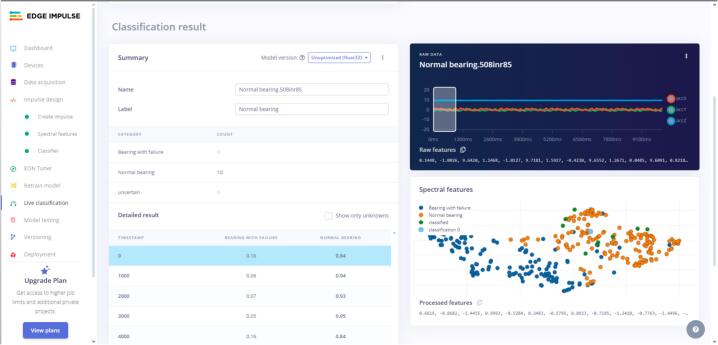
Live classification results.

A sample (default of 10 s) is taken to capture vibration data. The platform processes the information through the model and returns the results. Fig. 36 provides more details of the results.

In the following video, the validation process can be observed, which includes testing from data collection to deployment on an embedded device (Arduino Nano 33 BLE Sense) and MPU6050 with ESP32: https://youtu.be/R4zRcM2mTus.

### 7.1 Capabilities

• Recreate a real-life scenario focused on classrooms and practice ML and AI concepts on embedded devices.
• Easy to assemble.
• Easy to use.
• Simple and intuitive creation of classification models.
• Low-cost components.
• The software used has extensive documentation and tutorials.

### 7.2 Limitations

• Using online software to collect data and create and train the AI model can be problematic for users with limited internet access.
• Connecting other sensors and boards will require different configurations (connections, Arduino IDE codes for data capture, etc.).

## CRediT authorship contribution statement

**Andres Felipe Cotrino Herrera:** Writing – review & editing, Writing – original draft, Validation, Software, Methodology, Investigation, Formal analysis, Conceptualization. **Jesús Alfonso López Sotelo:** Writing – review & editing, Supervision, Conceptualization. **Juan Carlos Blandón Andrade:** Writing – review & editing, Supervision. **Alonso Toro Lazo:** Writing – original draft, Supervision.

## Declaration of competing interest

The authors declare that they have no known competing financial interests or personal relationships that could have appeared to influence the work reported in this paper.
